# Glioma Cell Proliferation Controlled by ERK Activity-Dependent Surface Expression of PDGFRA

**DOI:** 10.1371/journal.pone.0087281

**Published:** 2014-01-29

**Authors:** Dongfeng Chen, Duo Zuo, Cheng Luan, Min Liu, Manli Na, Liang Ran, Yingyu Sun, Annette Persson, Elisabet Englund, Leif G. Salford, Erik Renström, Xiaolong Fan, Enming Zhang

**Affiliations:** 1 The Rausing Laboratory, Department of Neurosurgery, Lund University, Lund, Sweden; 2 Beijing Key Laboratory of Gene Resource and Molecular Development, Laboratory of Neuroscience and Brain Development, Beijing Normal University, Beijing, China; 3 Department of Pathology, Lund University, Lund, Sweden; 4 Department of Clinical Science, Lund University, Malmö, Sweden; University of Florida, United States of America

## Abstract

Increased PDGFRA signaling is an essential pathogenic factor in many subtypes of gliomas. In this context the cell surface expression of PDGFRA is an important determinant of ligand sensing in the glioma microenvironment. However, the regulation of spatial distribution of PDGFRA in glioma cells remains poorly characterized. Here, we report that cell surface PDGFRA expression in gliomas is negatively regulated by an ERK-dependent mechanism, resulting in reduced proliferation of glioma cells. Glioma tumor tissues and their corresponding cell lines were isolated from 14 patients and analyzed by single-cell imaging and flow cytometry. In both cell lines and their corresponding tumor samples, glioma cell proliferation correlated with the extent of surface expression of PDGFRA. High levels of surface PDGFRA also correlated to high tubulin expression in glioma tumor tissue *in vivo*. In glioma cell lines, surface PDGFRA declined following treatment with inhibitors of tubulin, actin and dynamin. Screening of a panel of small molecule compounds identified the MEK inhibitor U0126 as a potent inhibitor of surface PDGFRA expression. Importantly, U0126 inhibited surface expression in a reversible, dose- and time-dependent manner, without affecting general PDGFRA expression. Treatment with U0126 resulted in reduced co-localization between PDGFRA and intracellular trafficking molecules e.g. clathrin, RAB11 and early endosomal antigen-1, in parallel with enhanced co-localization between PDGFRA and the Golgi cisternae maker, Giantin, suggesting a deviation of PDGFRA from the endosomal trafficking and recycling compartment, to the Golgi network. Furthermore, U0126 treatment in glioma cells induced an initial inhibition of ERK1/2 phosphorylation, followed by up-regulated ERK1/2 phosphorylation concomitant with diminished surface expression of PDGFRA. Finally, down-regulation of surface PDGFRA expression by U0126 is concordant with reduced glioma cell proliferation. These findings suggest that manipulation of spatial expression of PDGFRA can potentially be used to combat gliomas.

## Introduction

Gliomas are the most common primary tumors in adult central nervous system [1]. Intensive efforts in mechanistic and clinical investigations have significantly improved our understanding of the cellular and genetic basis of gliomas, however, the prognosis of glioma patients remains poor [2]. Patients with glioblastoma multiforme (GBM) have a survival time of only up to 2 years after diagnosis [3]. Low-grade gliomas will eventually progress into high-grade gliomas, although with varying kinetics among the patients. Understanding the mechanisms of glioma pathogenesis is crucial for the identification of new therapeutic targets to combat gliomas [4].

The activity of receptor tyrosine kinase (RTK) plays an important role in cell fate decision, cell proliferation and migration during neural development and adult neurogenesis [5]–[7]. Signaling of PDGRA is crucial for the pathogenesis of a substantial fraction of gliomas. PDGFRA is a characteristic marker of oligodendrocyte progenitor cells (OPCs) [5], [8]–[11], its disruption results in diminished oligodendrogenesis [12]. Previous studies have shown that PDGFRA is overexpressed in 30% of human gliomas, and that enhanced PDGFRA expression is essential in mouse glioma models [2], [13]–[15]. In particular, autocrine stimulation of PDGFRA signaling is suggested to be important for glioma initiation and progression [14], [16]. In mice, PDGFB overexpression in neonatal CNS or adult CNS, either by the transgenic approach on a p53^−/−^ background [17], or by the retroviral gene transfer approach [16], [18], [19], generated glioma-like tumor growth. Interestingly, tumors generated in these models all expressed PDGFRA [17], [18], [20]. Inhibition of PDGFRA signaling resulted in a reversion of transformed phenotype in glioma cell lines [21], or a reversion from high-grade to lower grade tumor histology in mouse model [22]. Several reports including ours suggest that human gliomas with high levels of PDGFRA expression are associated with frequent *IDH1* mutation, deletion of chromosome 1p and 19q, G-CIMP or proneural phenotype, infrequent EGFR amplification, younger age at disease diagnosis and better survival compared to other gliomas with lower levels of PDGFRA expression, but high levels of EGFR expression [23]–[26]. Thus, gliomas with high levels of PDGFRA expression and gliomas with high levels of EGFR amplification and expression may originate from different cellular and genetic origins [27]–[33].

Compared to the established close association between EGFR activation and *EGFR* gene amplification and mutation [34], the amplification, rearrangement and mutation of PDGFRA gene is present only in a small fraction of gliomas [35]–[38]. PDGFRA activation is primarily ligand-driven [2], [39], [40] and regulated by extracellular heparin sulfate proteoglycans [41]. The ligand-dependent PDGFRA signaling activity is to the first line controlled by the display of PDGFRA on cell surface to sense the microenvironment, and by the trafficking process of PDGFRA to control the duration and amplitude of signaling activities following ligand stimulation. Therefore, intracellular trafficking may critically control the activity of PDGFRA signaling.

Signaling of PDGFRA or other RTKs results in activation of Ras-Raf-MEK-ERK pathway in gliomas [42]. In addition, activation of Ras-Raf-MEK-ERK pathway in glioma can also be caused by genomic alterations in the components of Ras-Raf-MEK-ERK pathway [43]. Here we report that the cell surface expression of PDGFRA is negatively controlled by ERK activity, which has consequences for cell proliferation. Treatment of PDGFRA expressing glioma cells with MEK inhibitor U0126 [44], [45] resulted in a transient decline of ERK phosphorylation, followed by up-regulated phosphorylation of ERK. Up-regulated ERK phosphorylation is associated with a reduction of surface PDGFRA expression and a decline of glioma cell proliferation. Our characterization of PDGFRA trafficking through early endosome, recycling endosome and Golgi network suggests that diminished surface expression of PDGFRA following U0126 treatment was a consequence of a depletion of PDGFRA from endocytotic and recycling compartment, concomitant with enrichment of PDGFRA in the Golgi apparatus. U0126 mediated down-regulation of PDGFRA surface expression correlated with diminished cell proliferation. Our findings suggest that the trafficking of PDGFRA in glioma cells is regulated by MEK and ERK activity and can potentially be manipulated to combat glioma growth.

## Results

### Correlation between PDGFRA Surface Expression and Cell Proliferation in Glioma Cells

**Figure 1:**
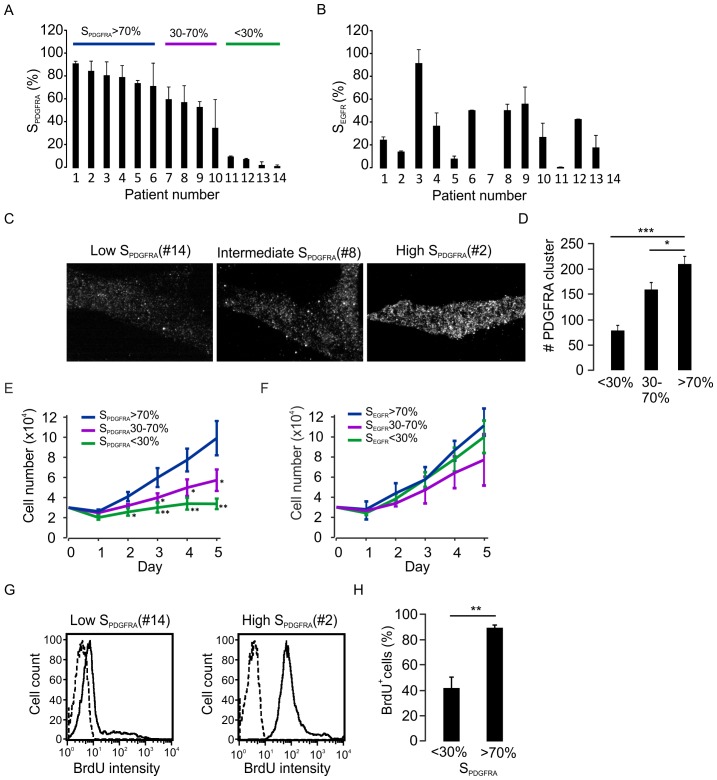
Correlation between PDGFRA surface expression and cell proliferation in human primary glioma cells. A. PDGFRA surface intensity was determined by FACS in glioma cell lines from 8 glioblastomas and 6 grade II astrocytomas. The glioma cell lines were divided into three subgroups according to the level of PDGFRA surface expression. The data represent mean±SD of the percentages of cells with surface PDGFRA expression from three independent FACS experiments. B, the same as A, but EGFR surface expression was assessed. C. The PDGFRA surface expression was detected using TIRFM. The representative images indicate the surface expression of PDGFRA clusters in a single glioma cell. D. The averages of PDGFRA clusters assessed in TIRFM were summarized in the three groups of cell lines. *p<0.05, ***p<0.001 (ANOVA, F-test, n = 14). E. Glioma cell proliferation profiles during 5 days *in vitro* culture were plotted against the extent of PDGFRA surface expression. *p<0.05, **p<0.01 (ANOVA, F-test, n = 14). F, Same as E, but the proliferation profiles were plotted against the extent of EGFR surface expression. G and H, more BrdU positive cells were detected in glioma cells with high PDGFRA surface expression. **p<0.01 (Student’s *t*-test, n = 8, glioma cell lines from #1, #2, #3 and #5 were in the >70% group and #11, #12, #13 and #14 in the <30% group.).

Using newly established glioma cell lines isolated from 8 glioblastomas and 6 grade II astrocytomas **(Table S1)**, we have assessed glioma cell proliferation in the context of PDGFRA expression on cell surface. No detectable amplification of the *PDGFRA* gene was observed in these cell lines [23]. We first used flow cytometry to compare the extent of surface PDGFRA expression in these cell lines. Interestingly, the cohort can be distinguished into three groups according to PDGFRA surface expression **(** **Figure 1A** **)**. These groups did, however, not exhibit any correlation with the extent of EGFR surface expression **(** **Figure 1B** **)**. The three groups were confirmed by total internal reflection fluorescence microscopy which measures the expression of PDGFRA in the immediate proximity (100–200 nm) of the plasma membrane **(** **Figure 1C and 1D** **)**. Using both approaches, three groups of glioma cells could be clearly distinguished with high, intermediate or low PDGFRA expression on the surface. Interestingly, the glioma cells with high surface expression of PDGFRA showed higher proliferation rates compared with those with lower surface expression of PDGFRA **(** **Figure 1E** **)**. Under our conditions, a correlation between surface expression of EGFR and cell proliferation rate was not detected **(** **Figure 1F** **)**. Furthermore, the high cell proliferation rates in glioma cells with high surface PDGFRA expression was confirmed using a BrdU incorporating approach (**Figure 1G and 1H**).

**Figure 2:**
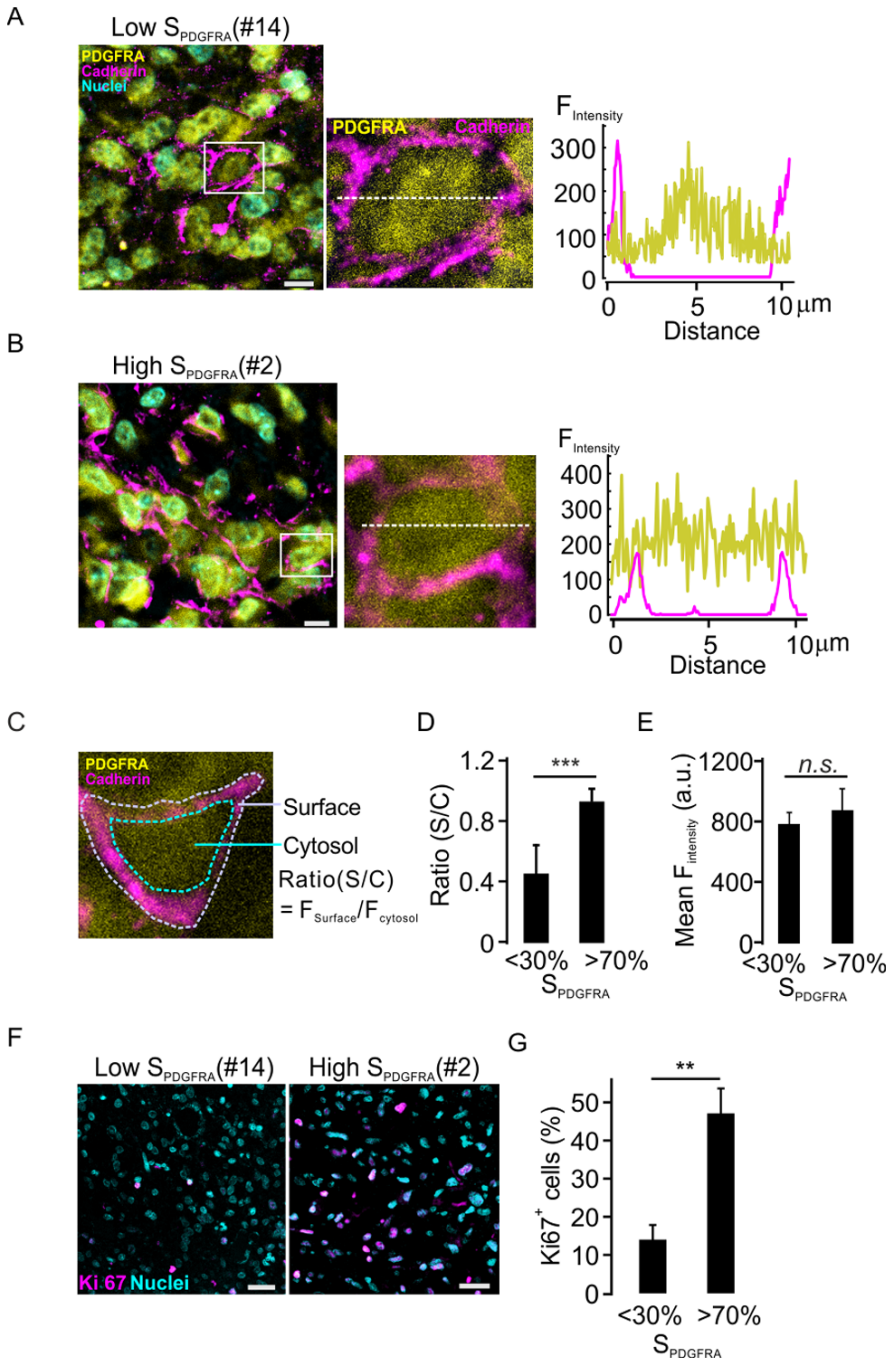
High-density surface expression of PDGFRA in glioma samples is associated with high cell proliferation rates *in vivo.* A. Left panel: representative confocal images of PDGFRA immunohistochemistry staining in glioma samples corresponding to the cell lines with low surface PDGFRA expression as in Figure 1A. Right panel: intensity profile along the white spot line in the middle image, zoom-in from box in left picture. B. Same as A, but sections from glioma samples corresponding to the cell lines with high surface PDGFRA. C. Depiction of the ratio between the mean PDGFRA fluorescence intensity of cell surface (I_surface_) to that of the cytosol (I_cytosol_). The surface area of PDGFRA fluorescence was defined by the staining of cadherin, a plasma membrane marker. D. Average ratios of PDGFRA expression in glioma tissues with high (#2, #3 and #5) or low PDGFRA surface expression (#12, #13 and #14). For each patient’s tissue, 14 cells were chose for the ratio quantitative analysis, n = 6, ***p<0.001 (ANOVA, F-test). E. Mean fluorescence intensity of PDGFRA in the whole cells was detected in tissues with low and high surface expression of PDGFRA. *n.s.*: no significance by Student’s *t*-test. 19 cells in each of tissues as in D were used for analysis. F. Representative confocal images of Ki-67 immunostaining in the indicated glioma samples. G. Average percentages of Ki67 positive cells under the conditions as in E. n = 3. **p<0.01 (ANOVA, F-test).

To estimate the surface PDGFRA expression in glioma cells in primary tissues, sections from the formalin-fixed and paraffin-embedded glioma samples derived from the same surgical resections as those for the generation of the cell lines described above were stained for PDGFRA in combination with the plasma membrane marker cadherin. In sections from samples corresponding to the cell lines with low surface PDGFRA expression as shown in Figure 1A, low surface expression of PDGFRA was also detected in primary tissues **(** **Figure 2A** **)**. Concordantly, sections from samples corresponding to those cell lines with high surface PDGFRA expression *in vitro* showed high surface expression of PDGFRA **(** **Figure 2B** **)**. As shown in **Figure 2C**, the surface region was clearly defined by plasma membrane marker cadherin, the ratio between PDGFRA mean intensity in the surface region and that in the cytosol was about two-fold higher in samples with the corresponding cell lines showing high surface PDGFRA expression, when compared with samples with associated cell lines showing low surface PDGFRA expression (0.94±0.08 versus 0.46±0.18, *p<0.001, n = 6*, **Figure 2D**). However, the mean fluorescence intensity of PDGFRA in the whole cells was not significantly different between the glioma tissues with low or high surface PDGFRA tissues (**Figure 2E**). Interestingly, immunohistochemistry staining of the proliferation marker Ki67 showed that the glioma cell proliferation was about three-fold higher in tissues with high surface PDGFRA expression compared with those with tissues with low surface PDGFRA expression (*p<0.001, n = 6*, **Figure 2F and 2G**). These findings suggest a strong correlation between surface expression of PDGFRA and cell proliferation in glioma cells.

### Requirement of Cytoskeleton Support for PDGFRA Surface Expression

**Figure 3:**
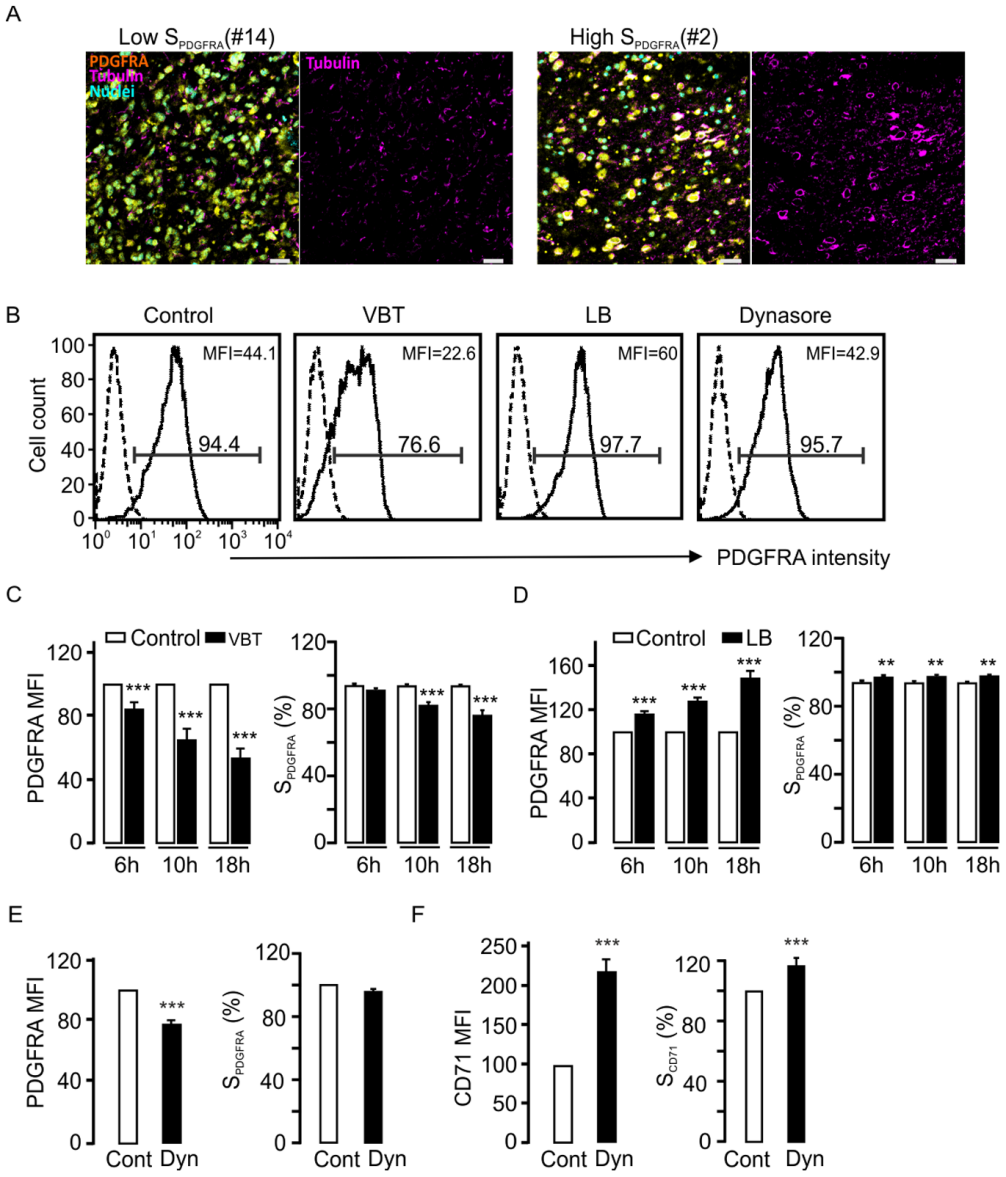
PDGFRA surface expression is controlled by cytoskeleton system. A. Representative confocal images of tubulin immunostaining in the glioma tissue sections with high (n = 3, tissue #2, #3 and #5) or low (n = 3, tissue #12, #13 and #14) surface PDGFRA expression. B. Representative FACS histograms depict the effects of pharmacological inhibitors VBT (500 nM), LB (500 nM) and Dyn (40 µM) on surface PDGFRA expression in cell lines #1, #2 and #3. C. Time dependent decreases of surface PDGFRA expression measured by PDGFRA intensity (MFI, left) and the percentage of PDGFRA-positive cells (right) after treatment of VBT (n = 3, the same cell lines were used as in B. p<0.001, Student *t*-test). D. Increases of surface PDGFRA expression by intensity (left) and percentage (right) after treatment of LB. E. No significance change in the percentage of surface PDGFRA-positive cells but decrease in the surface PDGFRA intensity after dynasore treatment. (n = 3, the same cell lines were used as in B, p<0.001 for MFI, *t*-test). F. Increase of CD71 surface expression after dynasore treatment (n = 3, the same cell lines were used as in B, p<0.001, *t*-test).

Surface expression of receptors is typically regulated by endosomal intracellular trafficking processes, for which the cytoskeleton is essential [46], [47]. To address this possibility, we next performed experiments to investigate whether the spatial distribution of PDGFRA is dependent on the cytoskeleton. Immunohistochemical staining in glioma tissues showed that gliomas with high surface PDGFRA expression presented higher expression of β-tubulin than gliomas with low surface PDGFRA expression **(** **Figure 3A** **)**. These results indicate that inhibition of β-tubulin might be a way to decrease the surface of PDGFRA expression. To observe the effects of cytoskeleton on surface PDGFRA expression in living glioma cells, we disrupted cytoskeletal organization by pharmacological inhibitors in the glioma cell lines #1, #2 and #3 with high surface PDGFRA expression **(** **Figure 3B** **)**. We observed a time-dependent decrease of surface PDGFRA expression after treatment with tubulin inhibitor Vinbastine (VBT). After 18 hrs treatment, a significant decrease in surface PDGFRA expression was evident, as assessed by both the intensity of PDGFRA expression (as measured in mean fluorescence intensity (MFI)), and the percentages of the cells with surface PDGFRA expression **(** **Figure 3C** **)**. However, the intensity of surface PDGFRA, rather increased after treatment with latrunculin B (LB), an actin polymerization inhibitor **(** **Figure 3D** **)**. To further identify whether the endocytotic pathway controls PDGFRA surface expression, we treated cells with dynasore (Dyn) to inhibit dynamin-dependent scission of newly formed endocytotic vesicles from the plasma membrane [48]. Dyn-treatment resulted in a decrease in the intensity of surface PDGFRA expression, without measurable changes in the percentages of PDGFRA-positive cells **(** **Figure 3E** **)**. As a positive control, surface expression of CD71, a representative of receptor-activated endocytosis, was increased in both intensity of surface expression and percentages of the cells with surface expression after Dyn treatment **(** **Figure 3F** **)**. Taken together, these findings suggest that surface PDGFRA expression is regulated by cytoskeleton-dependent and non-receptor activated endocytotic pathway.

### Regulation of PDGFRA Surface Expression by ERK Activity

**Figure 4:**
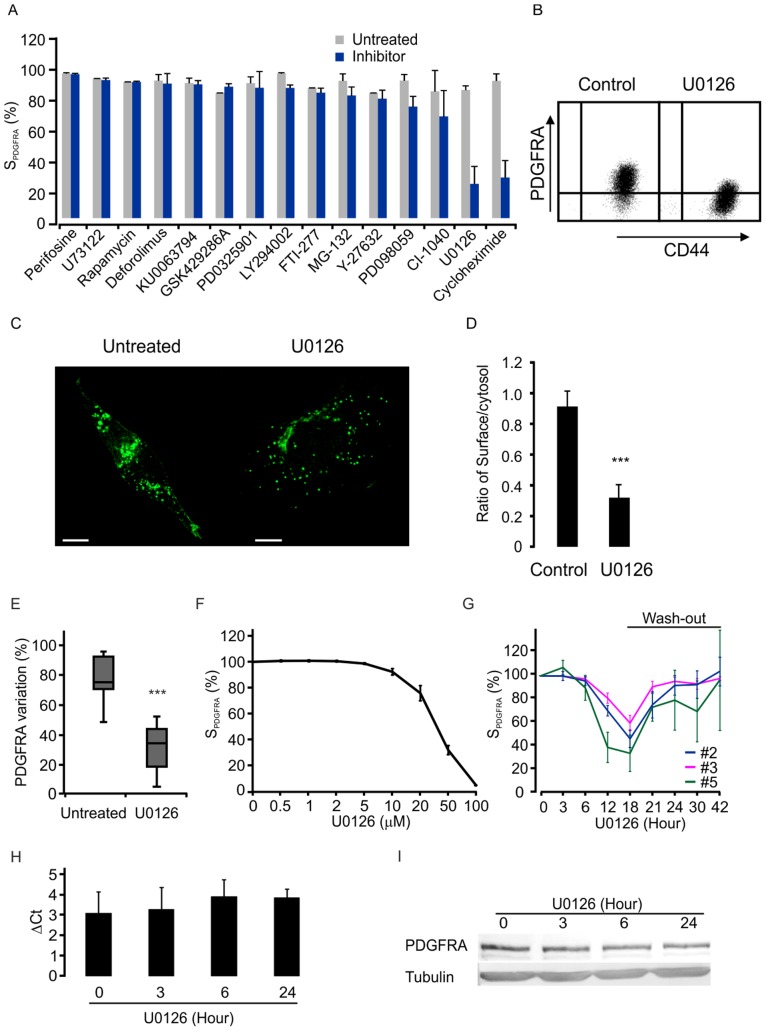
PDGFRA surface expression is down-regulated by MEK inhibitor U0126. A. The inhibitors were screened based on their capacity to down-regulate surface expression of PDGFRA in glioma cells. B. MEK inhibitor U0126 decreased PDGFRA surface expression detected by FACS in cell line #2. C. Representative confocal images showed PDGFRA expression with or without U0126 treatment in cell line #2. D. Average ratios of surface and cytosol PDGFRA expression in a glioma cell line with or without U0126 treatment in cell line #2. 18 cells were used for analysis in each group, ***p<0.001, *t*-test. E. The effect of U0126 on PDGFRA surface expression detected by FACS in 9 cell lines (cell lines #1–7, #9 and #14, p<0.001, *t*-test). F. Dose-dependent effects of U0126 on surface expression of PDGFRA in glioma cells. Data showed as average ± SD. G. Reversible time-dependent effect of U0126 on the PDGFRA expression from the three indicated cell lines. H and I. No significant effect of U0126 on PDGFRA expression at the mRNA and protein levels as analyzed in cell line #2. ΔCt was calculated by the mean Ct value of PDGFRA subtracted with the mean Ct value of GAPDH. The data are presented by average ± SEM of 5 repeats in 3 passages cells. The western blots are representative of three independent replicates. These experiments were performed with the cell line #2.

PDGFRA activation triggers the activation of Ras-Raf-MEK-ERK and PI3K-AKT pathways. Both Ras-Raf-MEK-ERK and PI3K-AKT pathways are highly active in gliomas [43], [49]. Using a panel of small molecule inhibitors, we tested whether the activity of Ras-Raf-MEK-ERK cascade and PI3K-AKT pathway could regulate surface PDGFRA expression in the glioma cell lines #1, #2 and #3 with high surface PDGFRA expression. We identified U0126, a specific inhibitor for MEK activity, as a strong and reversible inhibitor of PDGFRA surface expression. LY290042, a PI3K inhibitor, produced a very weak similar response, whereas all other inhibitors tested were ineffective, including PD0325901, PD098059 and CI-1040 (MEK inhibitors), Perifosine (AKT inhibitor), KU-0063794, Deforolimus and Rapamycin (mTOR inhibitor) **(** **Figure 4A** **)**. As representatively illustrated in **Figure 4B**, FACS data showed that U0126 treatment resulted in reduced cell surface expression of PDGFRA in the glioma cell line #2, but not CD44, a cell-surface receptor for hyaluronic acid. In agreement with this, confocal image analysis also showed that in cells untreated with U0126, the majority of PDGFRA molecules were localized in the plasma membrane with focal clustering. In U0126 treated cells, the majority of the PDGFRA molecules were dispersed in the cytosol (**Figure** **4C and 4D**). Moreover, this U0126 induced reduction of surface PDGFRA occurred in all tested glioma cell lines **(** **Figure 4E** **)**.

U0126 dependent decline of surface PDGFRA expression was dose-dependent with significant effects evident within the range of 10 to 50 µM as shown in the glioma cell line #2 with high surface PDGFRA expression **(** **Figure 4F** **)**. Timewise, this effect was first evident after 6 hrs treatment **(** **Figure 4G** **)**. After 18 hrs U0126 treatment, the surface PDGFRA expression decreased to about 10% of that in control cells **(** **Figure 4G** **)**. This decrease of surface PDGFRA expression persisted in the presence of U0126 treatment. However, following withdrawal of U0126, surface PDGFRA expression was significantly recovered. Furthermore, using real-time RT-PCR and western-blot analyses in the glioma cell line #2 with high surface PDGFRA expression, we demonstrated that in cells with significant decline of the surface PDGFRA expression, no measurable reduction of PDGFRA transcript and protein was detected (**Figure 4H and 4I** **)**.

**Figure 5:**
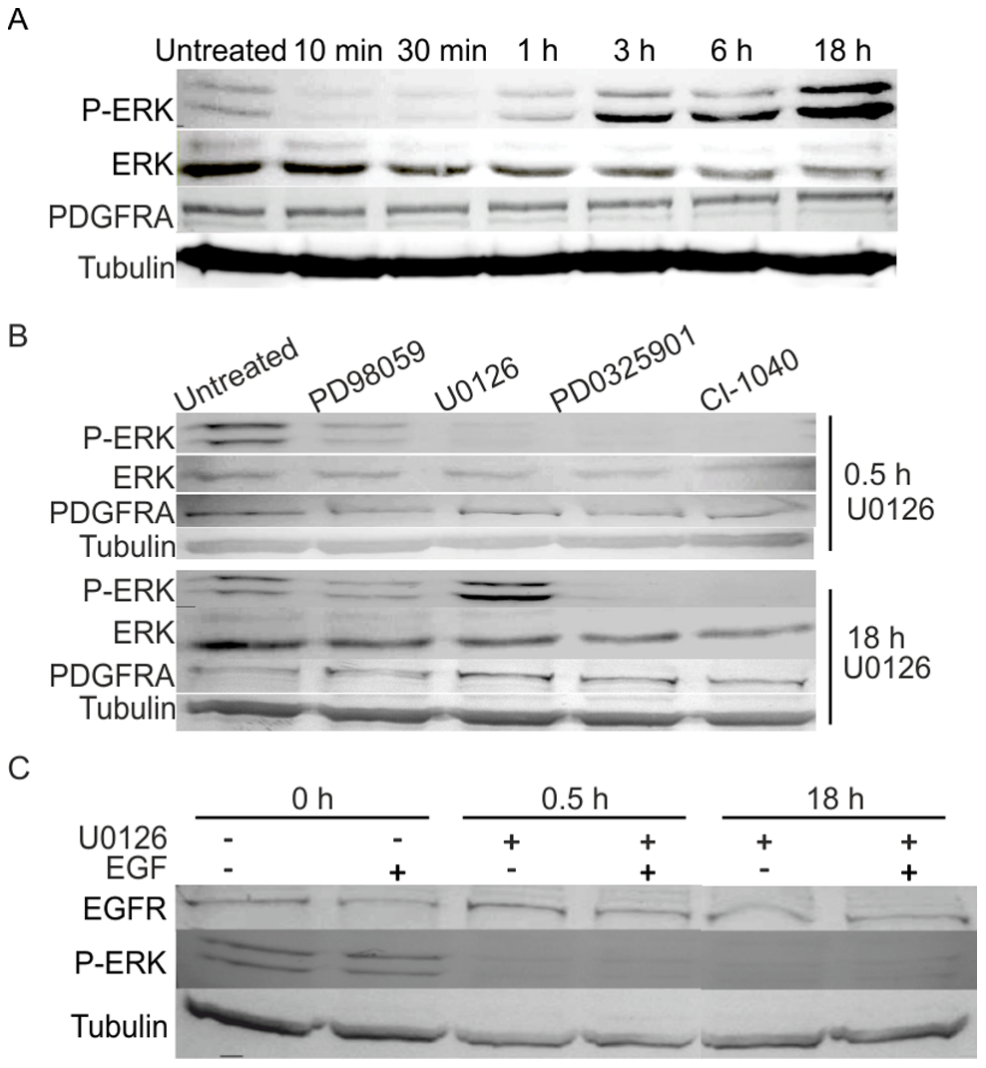
U0126 induced down-regulation of PDGFRA surface expression is concordant with a positive feedback of ERK activity. A. Represent immunoblots of ERK activities at different time points following U0126 treatment. The blot is representative from 3 independent experiments. B. Represent immunoblots of ERK activities after 0.5 or 18-free A549 cells. All the experiments were performed in the cell lines #1, #2 and #3.

To investigate the pathway involved in U0126-dependent reduction of surface PDGFRA expression on glioma cells, we focused on the ERK pathway since a series of studies reported that U0126 inhibits the phosphorylation of ERK by MEK [44], [45]. Under our conditions, U0126 treatment caused an initial decline in ERK phosphorylation, followed by an unexpected increase in ERK phosphorylation between 3 and 18 hrs of U0126 treatment in the glioma cell lines #1, #2 and #3 with high surface PDGFRA expression **(** **Figure 5A** **)**. The other MEK inhibitors e.g. PD98059, PD325901 and CI-1040, that did not (or only weakly) affect surface PDGFRA expression **(** **Figure 4A** **)**, completely blocked ERK phosphorylation during the entire 18-hr treatment **(** **Figure 5B** **)**. Moreover, in PDGFRA-free A549 cell lines, ERK phosphorylation was inhibited during treatment with U0126 for 18 hrs **(** **Figure 5C** **)**. These findings suggest that U0126 induces a positive feedback of ERK phosphorylation in glioma cells is required for the down-regulation of surface PDGFRA expression. These findings also suggest that PDGFRA expression is required for U0126 to exert a positive feedback on ERK phosphorylation, which in turn is required for the down-regulation of PDGFRA expression in the glioma cell surface.

### Reduced Dwelling of PDGFRA in Intracellular Trafficking System following U0126 Treatment

**Figure 6:**
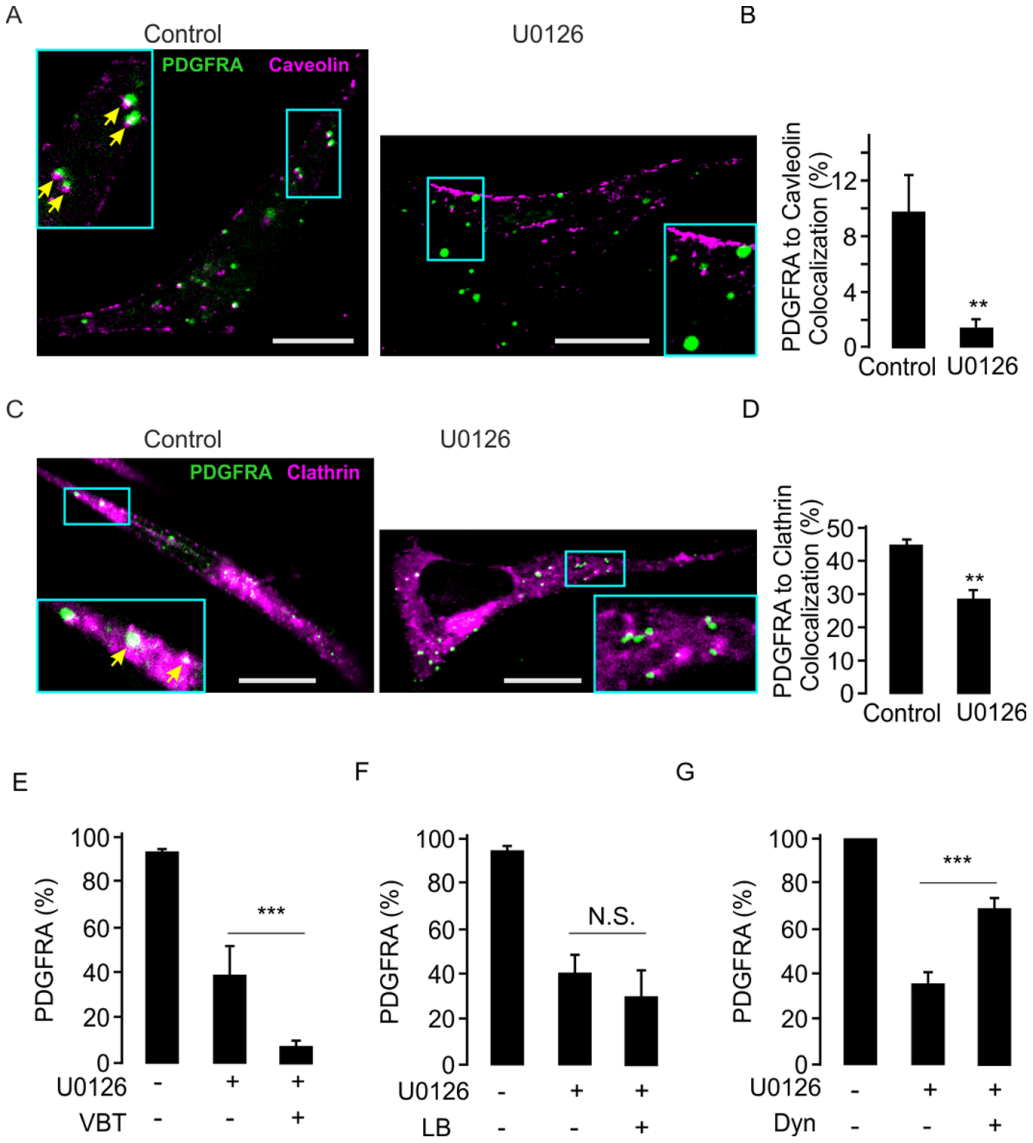
PDGFRA was deviated from intracellular trafficking system after treatment with U0126 for 18 A. Immunostaining images of PDGFRA and caveolin in cell line #2 following U0126 treatment. The large inserted picture is the zoom-in from the small box. Arrows indicate the colocalized clusters. B. Average PDGFRA co-localization to caveolin detected under the same conditions as in A (12 cells in each treatment, **p<0.01, *t*-test). C. Immunostaining of PDGFRA and clathrin in the cell line #2 with or without U0126 treatment. Arrows indicate the colocalized clusters. D. Average PDGFRA co-localization to clathrin detected under the same conditions as in C (12 cells in each treatment, **p<0.01, *t*-test). E. FACS data show PDGFRA surface expression after treatment of U0126 in combination with VBT in the glioma cell lines #2, #3 and #5 (n = 3, p<0.001, *t*-test). F. No effect of LB on U0126 induced reduction of PDGFRA surface expression in the same cell lines as in E (n = 3, p>0.05, *t*-test). G. Counteraction of U0126 effects on PDGFRA surface expression by dynasore treatment in the same cell lines as in F (n = 3, p<0.001, *t*-test).

We next hypothesized that the endocytotic pathway is involved in U0126-dependent reduction of surface PDGFRA expression. The endocytotic processes can be subdivided into two gross categories, caveolin- and clathrin dependent endocytosis [50]. Confocal immunostaining analyses showed that about 10% of PDGFRA co-localized with caveolin-1, even though the protein is lowly expressed in glioma cells. Intriguingly, U0126 treatment decreased the co-localization of PDGFRA and caveolin-1 from 10% to about 2%–3% in the glioma cell line #2 **(** **Figure 6A and 6B** **)**. The other endocytotic protein clathrin was more highly expressed and, indeed, most of the PDGFRA co-localized with this protein in untreated cells. Interestingly, co-localization of PDGFRA to clathrin was also significantly reduced by U0126 treatment in the same cell line (**Figure 6C and 6D**). These results suggest that U0126 induced reduction of surface PDGFRA expression is concomitant with a deviation of PDGFRA from the endocytotic pathways.

To identify which trafficking molecules that are required for U0126 dependent reduction of surface PDGFRA expression, we combined U0126 treatment with pharmacological inhibitors towards different trafficking molecules. Firstly, combined treatment with U0126 and VBT resulted in a synergistic effect in the reduction of cell surface expression of PDGFRA compared to U0126 treatment alone in the glioma cell lines #2, #3 and #5 with high surface PDGFRA expression (**Figure 6E** **)**. Secondly, actin depolymerization by LB treatment (0.5 µM for up to 18 hrs) did not alter the effect of U0126 on surface PDGFRA expression (**Figure 6F** **)**. Most intriguingly, Dyn treatment significantly counteracted U0126-dependent of PDGFRA internalization (**Figure 6G** **)**. These results further confirmed that endocytotic pathway is required for the maintenance of PDGFRA surface expression.

**Figure 7:**
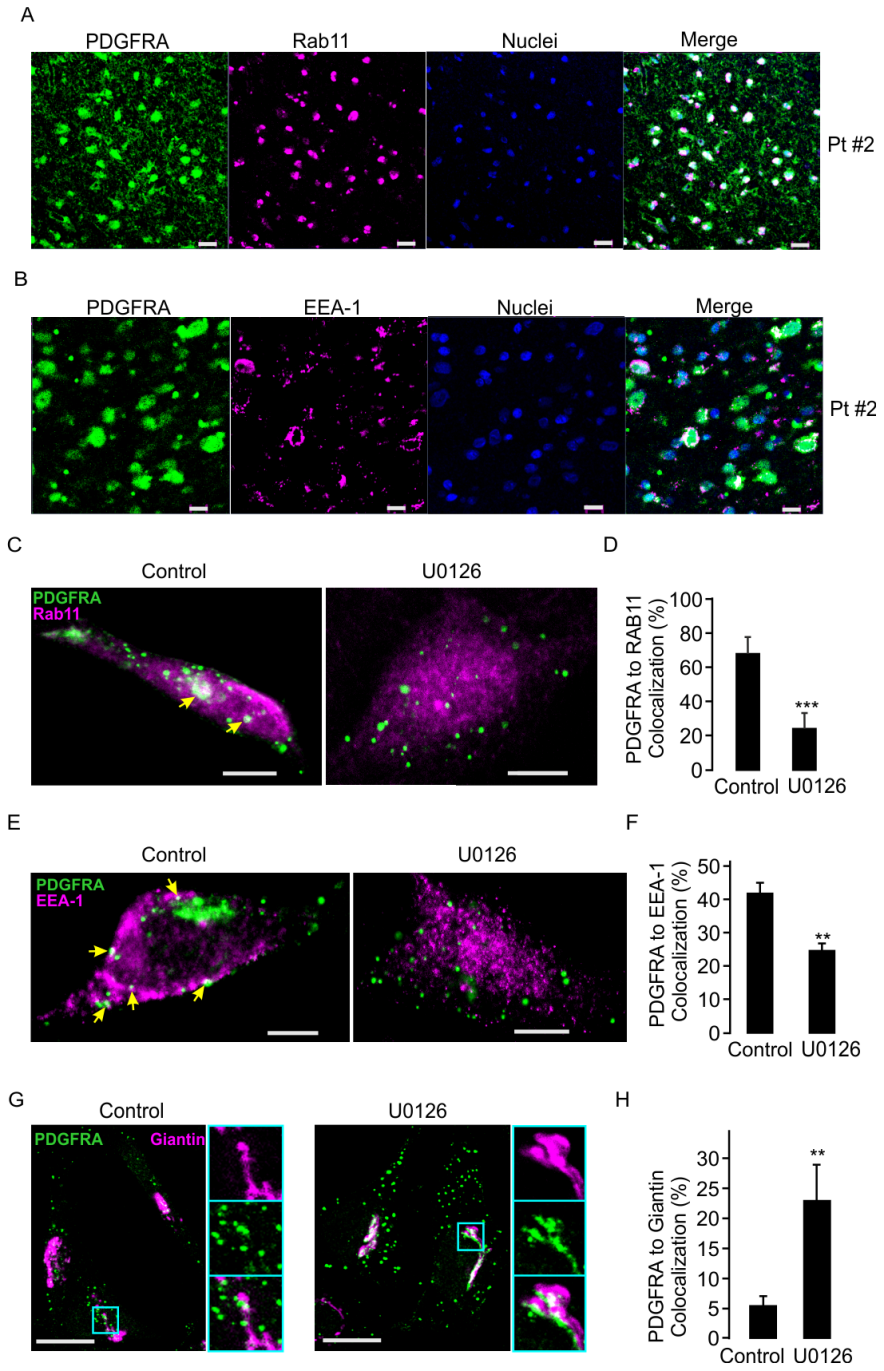
U0126 treatment reduces the dwelling of PDGFRA in recycling system. A. Representative confocal images of PDGFRA and Rab11 staining in glioma tissues of sample #2. B. The same condition as in A, but EEA-1 was detected. C. Immunostaining of PDGFRA and Rab11 with or without U0126 treatment in cell line #2. Arrows indicate the colocalized clusters. D. Average of PDGFRA co-localization to Rab11 detected under conditions as in C (18 cells in each condition, ***p<0.001, *t*-test). E. Immunostaining of PDGFRA and EEA-1 before and after U0126 treatment in cell line #2. Arrows indicate the colocalized clusters. F. Average of PDGFRA co-localization to EEA-1 under the same conditions as in E (28 cells in each condition, **p<0.01, *t*-test). G. Same as in E but Giantin was detected. The three separated images (right), the top, middle and bottom indicating Giantin, PDGFRA and respectively, are the zoom-in from box in left picture. H. Average of PDGFRA co-localization to Giantin under the same conditions as in G (42 cells in each condition. **p<0.01, *t*-test).

Finally, we investigated whether the internalized PDGFRA can be transported into intracellular recycling system. Immunostainings in glioma tissues demonstrated that the endocytosis-recycling molecules Rab11 and EEA-1 are highly expressed in the glioma cell line #2 (**Figure 7A and 7B**). Furthermore, the staining of cultured single glioma cells also showed a high rate of co-localization of PDGFRA to Rab11 and to EEA-1 (**Figure 7C and 7E**). After U0126 treatment, the co-localization of PDGFRA to Rab11 and EEA-1 decreased significantly (**Figure 7D and 7F**), suggesting that PDGFRA is deviated from endocytosis-recycling system after U0126 treatment.

We further estimated the localization of PDGFRA molecules in relation to Giantin-marked Golgi network following U0126 treatment in the same cell line. Co-stainings of PDGFRA and Giantin showed that in untreated cells, a low proportion of intracellular PDGFRA resides in or close to Golgi network. Following U0126 treatment, co-localization of PDGFRA and Giantin was 4-fold increased, suggesting that PDGFRA translocates from the trafficking cargo system to the Golgi network (**Figure 7G and 7H**).

### Down-regulated Cell Proliferation by U0126 Treatment

**Figure 8:**
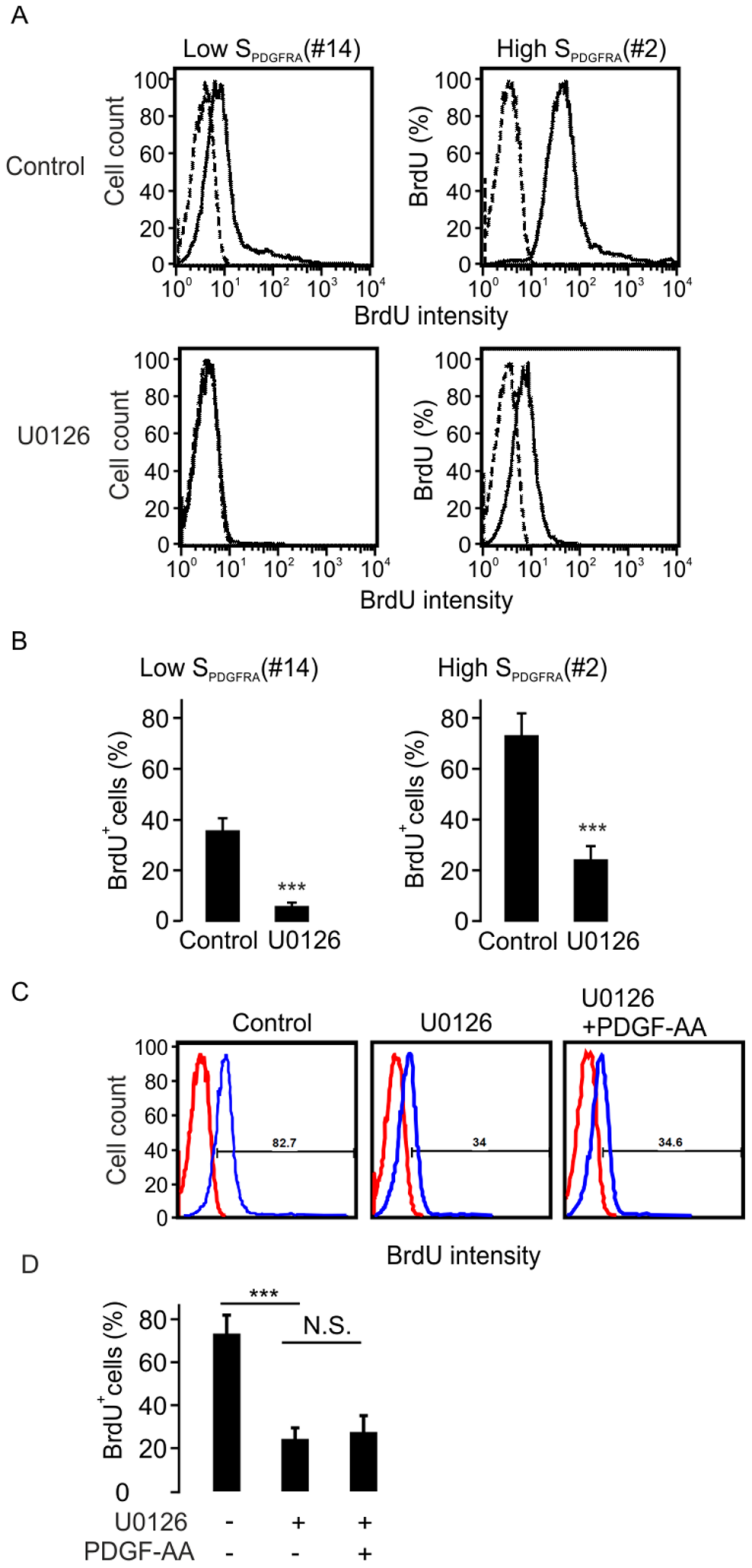
Down-regulation of glioma cell proliferation following U0126 treatment. A. FACS analysis showing BrdU positive glioma cells with low or high surface PDGFRA expression with or without U0126 treatment. B. Statistical analysis of BrdU positive cells under the same conditions as in A. The data were derived from three independent experiments (***p<0.001 ANOVA, F-test). C. Representative histograms showing the inhibition of PDGF stimulated glioma cell growth by U0126 treatment in cell line #2. D. Average percentages of BrdU positive cells analyzed under conditions as in C. n = 3 (cell lines #2, #3 and #5), ***p<0.001 (F-test).

Our findings show that glioma cell proliferation is associated with surface expression of PDGFRA, which in turn is efficiently decreased by U0126. This raises the question whether glioma cell proliferation can be regulated by U0126. To address this point, we measured cell proliferation by BrdU incorporation in glioma cells with low or high surface expression of PDGFRA (**Figure 8A**). After U0126 treatment for 18 hrs, the proliferation rates were significantly decreased in both cell lines. In the low-surface PDGFRA expressing cells, the percentage of BrdU positive cells decreased from 35.2±8.5% to 5.7±2.6% after U0126 treatment, and a similar effect was observed in high-surface PDGFRA expressing cells (from 73.6±14.8% to 24.5±8.9%; **Figure 8B**). To further address whether the activation of PDGFRA by PDGF can affect the U0126-blocked cell proliferation, we combined the treatment with U0126 and PDGF-AA in the cell lines #2, #3 and #5, and found that the PDGF-AA cannot revert the effect of U0126 on cell proliferation **(** **Figure 8C and 8D** **)**.

## Discussion

Aberrant PDGFRA signaling plays a central role in the pathogenesis of a high proportion of gliomas. Current studies demonstrate that this is caused by mechanisms such as PDGF autocrine or paracrine stimulation [14], [16], PDGFRA gene amplification or mutations [13], [35]–[38]. Our report extends on previous studies in that we demonstrate the importance of trafficking and surface expression of PDGFRA for glioma growth. Our findings show that also in the absence of acute ligand stimulation, PDGFRA molecules exhibit dynamic trafficking in glioma cells, and this trafficking process is regulated by MEK-ERK signaling. A surface location of PDGFRA is required for glioma cells to sense its microenvironment and down-regulation of surface expression of PDGFRA or other RTKs may be a successful approach to inhibit glioma cell growth.

To achieve a high degree of specificity of PDGFRA-mediated transmembrane signaling, controlled trafficking of PDGFRA between cytosol compartments and cell surface is important. Both the variations in the extent of surface expression of PDGFRA in glioma cells from different patients, as well as its regulation by MEK-ERK activity following U0126 treatment suggest that the PDGFRA spatial distribution could be regulated by cell signaling.

Previous reports have indicated the role of PDGFRA signaling in the activation of Ras-Raf-MEK-ERK pathway [14], [16], [35], [36], [51], [52], and aberrant activation of Ras-Raf-MEK-ERK pathway can be causal for gliomas [26], [49], [53]–[55]. PDGFRA signalling is likely dependent on PDGFRA endocytosis because endocytosis of receptor into endosomes (where the MEK signal molecules reside and ERK molecules are activated) is required for the activation of signaling [56]. Previous studies have demonstrated that RTK signaling depends on ligand-dependent RTK endocytosis processes [57]–[59]. However, our findings suggest a causal relationship in the reverse direction, with surface PDGFRA expression being regulated by the activity of MEK-ERK. Our results show that the number of PDGFRA molecules in plasma membrane decreases following enhanced ERK activity. Consequently, the ability of glioma cells to respond to their microenvironment may be blunted. Moreover, MEK and ERK are aberrantly active in gliomas and in many other cancers. Our findings indicate a new possibility that the trafficking of the receptors can be targeted by the signaling activities of the receptors.

**Figure 9:**
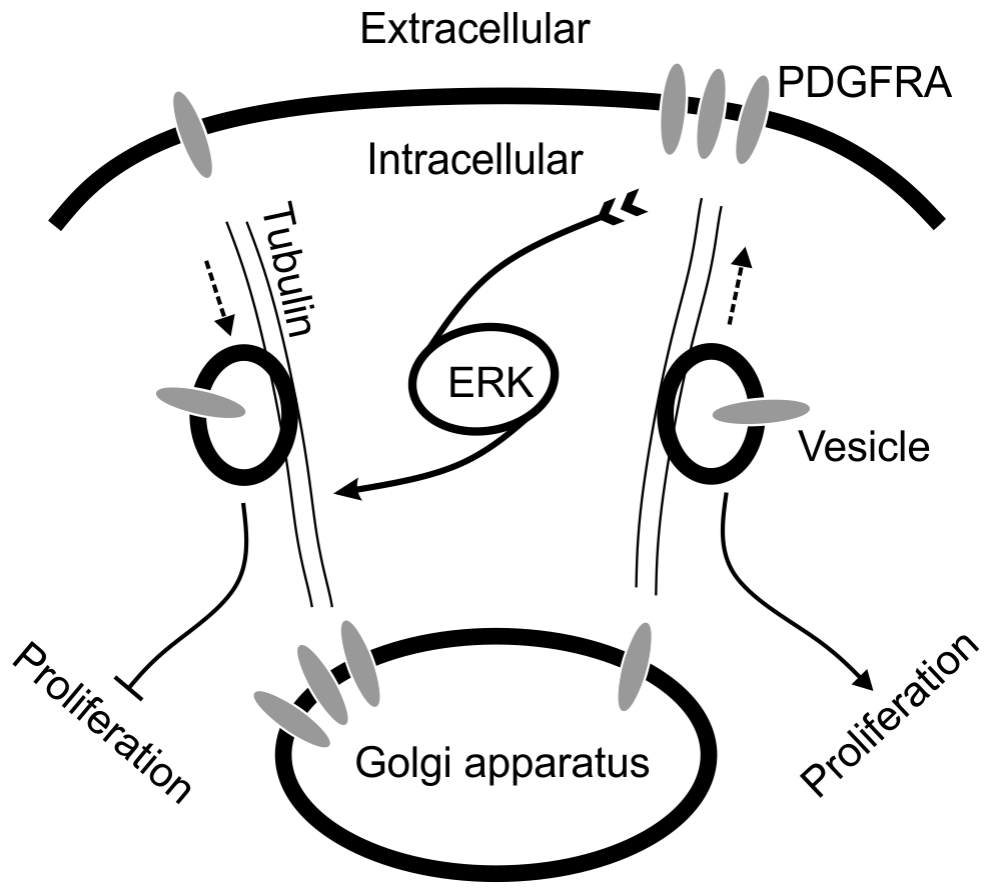
A carton illustrates association of surface PDGFRA with cell proliferation via ERK signaling pathway in glioma cells. Even in the absence of acute ligand stimulation, PDGFRA is constitutively, but slowly trafficking in glioma cells. In this trafficking process, PDGFRA is transported in vesicles as cargo along cytoskeleton highway. The extent of endocytosis and recycling of PDGFRA is sensed by the ERK activity. High ERK activity could lead to changes resulting in enhanced endocytosis but diminished recycling, with PDGFRA enriched in Golgi apparatus. As a consequence, surface PDGFRA expression is down-regulated. This process would dampen the ligand stimulation.

MEK might regulate PDGFRA surface expression via the activation of ERK molecules, which in turn translocate to the nucleus to activate RTK related transcriptional programs; surface expression of PDGFRA could be a consequence of the activated transcriptional programs [60]. In this scenario, PDGFRA surface expression increases in proportion to total PDGFRA expression, which is a both inefficient and slow. This is because a large proportion of newly synthesized PDGFRA molecules will spend considerable time to transverse the cytosol, a period during which they cannot affect signal transduction. Our findings suggest the existence of more efficient mechanism to regulate PDGFRA surface expression via controlling the intracellular trafficking system. Our data clearly show that the MEK inhibitor U0126 down-regulated surface PDGFRA expression within 6 hrs without noticeably changing total PDGFRA expression. Surprisingly, after an initial drop in ERK phosphorylation in response to U0126 treatment, a strong enhancement was seen after 18 hrs. This U0126 induced positive feedback of ERK activity has also been observed in hepatocellular carcinoma cells [61]. Moreover, our findings suggest that inhibition of MEK coupled with a strong positive feedback of ERK activity may in turn regulate steady-state RTK trafficking, resulting in a re-localization of PDGFRA from internalizing and recycling endosomes to the Golgi apparatus (**Figure 9**).

Previous reports demonstrate that RAS-PI3K, but not MEK, signaling regulates the trafficking of signal molecules between the cytoplasm and nucleus [62]. In contrast, MEK but not PI3K signals regulate cellular global trafficking events, i.e. tubulin nucleating [63], [64] or actin remodeling [65], [66]. Both these processes are of fundamental importance for trafficking of signal proteins between the plasma membrane and cytosolic compartments. Furthermore, our findings demonstrate that the MEK signaling could directly regulate the trafficking-related endocytotic and recycling processes. U0126 treatment significantly decreased co-localization of PDGFRA molecules to clathrin and caveolin, but increased PDGFRA localization to the Golgi apparatus, as assessed by co-localization with Giantin. This re-localization of PDGFRA coincided with diminished localization of PDGFRA to EEA1-positive early endosomes and RAB11-positive recycling endosomes. These results clearly show that positive feedback of ERK activity deviates PDGFRA from the intracellular recycling trafficking network to the Golgi apparatus. These findings are compatible with the localization and generation of Ras-Raf-MEK-ERK complexes in the early endosome compartment [67]. These results indicate an interesting scenario in that in addition to PDGFRA trafficking in gliomas, this mechanism might be applicable to the trafficking of other RTKs in other cancers, thereby providing a new approach to design cancer therapy strategy.

## Materials and Methods

### Ethics Statement

The collection and use of the human tissues in this study were performed after obtaining written consent from all participants, in accordance with a study protocol approved by The Regional Ethical Review Board in Lund, Sweden with the permission H15 642/2008. Glioma biopsies of fresh tumor tissue were obtained from patients operated at the Clinic of Neurosurgery, Lund University Hospital, Sweden. Glioma tissue blocks derived from surgical excision biopsies were obtained from the archive of Department of Pathology, Lund University Hospital.

### Inhibitors and Growth Factors

U0126, PD0325901, PD184352, PD98059, KU0063794, Deforolimus, Perifosine, LY294002, GSK429286A, Y-27632 MG-132 and rapamycin were purchased from Selleck Chemicals. FTI-277, U73122, Vinblastine (VBT), Latrunculin B (LB) and Dynasore (Dyn) were purchased from Sigma. PDGF-AA and FGF2 were purchased from PeproTech.

### Isolation and Culture of Glioma Cells

For preparing viable glioma cells, fresh specimen was cut into small pieces, and incubated in IMDM with 0.5 mg/ml collagenase (Sigma) and 25 mg/ml DNAse (Sigma) at 37°C for 40 minutes. Red cells were lysed with NH_4_Cl. The remaining glioma cells were washed in PBS containing 2% fetal calf serum (FCS). Glioma cells were grown in DMEM/F12 (1∶1) medium (Life Technologies) supplemented with 2% FCS, D-(+)-glucose solution (0.6%; Sigma), heparin (5 µg/ml; Sigma), sodium bicarbonate (0.1%; Sigma), B27 minus vitamin A supplement (Life Technologies), FGF2 (20 ng/ml; Peprotech).

### Flow Cytometric Analysis

About 10^5^ to 10^6^ cells were stained with allophycocyanin (APC)-conjugated anti-CD44 monoclonal antibody (clone C26, BD) in combination with phycoerythrin (PE)-conjugated mAb against PDGFRA (clone αR1, BD) or PE-conjugated mAb against CD71 (clone MEM-75, BioLegend) or PE-conjugated mAb against EGFR (clone EGFR.1, BD) for 10 minutes in the dark at 4°C. Subsequently, cells were washed once with PBS and suspended in 500 µl PBS supplemented with 2% FCS and 1.0 mg/ml 7- aminoactinomycin D (Sigma). The expression of indicated proteins was evaluated using a FACSCalibur (Becton Dickinson Immunocytometry Systems) and analyzed using FlowJo software.

### Real-time RT-PCR Analysis

Total mRNA from the glioma cells was prepared using RNAeasy kit (Qiagen) in accordance with the manufacture’s protocol. Twenty microliters of cDNA was synthesized using 1 µg total mRNA within SuperScript First-Strand Synthesis System (Life Technologies) according to the manufacturer’s instructions. The 10 µl total reaction volume included in 2.5 µl cDNA, 2.5 µl primers and 5 µl TaqMan MasterMix (Cat. NO.4304437, Life Technologies). The primers of PDGFRA (Cat NO. 4331182) and endogenous control gene GAPDH (Cat NO. 4333764T) were purchased from Life Technologies. The reaction was performed by the ViiA7 Fast Real-time PCR Systems (BD, USA) with the following protocol: after an initial denaturation step at 50°C for 2 minutes plus 94°C for 10 minutes, 40 cycles were performed at 95°C for 15 s and 60°C for 60 s. The speed of temperature changes was 1.6°C/s. The Ct value was measured by the software ViiA7 v1.2.2.

### Western Blot Analysis

Cells from patient No. 2 were treated with or without indicated inhibitors, washed with cold PBS and lysed in cell lysis buffer with a cocktail of protease inhibitors (Sigma) for 30 min on ice. Aliquots of 40 µg total proteins of lysed cells were separated with SDS-PAGE and transferred to PVDF membrane. The membrane was blocked with 3% BSA and 0.5% goat serum, and incubated with Rabbit anti-human PDGFRA monoclonal antibody (clone D1E1E, Cell Signaling) or Rabbit anti-human p44/p42 MAPK monoclonal antibody (clone 137F5, Cell Signaling) or Rabbit anti-human Phospho-p44/p42 MAPK monoclonal antibody (clone 20G11, Cell Signaling) at 4°C overnight. Subsequently, the membrane was washed with PBS and incubated with goat anti-mouse IgG-HPR and visualized with Opti-4CNTM substrate kit (BIO-RAD).

### Cell Proliferation Assay

Glioma cells were seeded into 24-well plate, 3×10^4^ cells/well. Cell number was countered every 24 hrs for 5 days. Cell surface PDGFRA expression and EGFR expression were evaluated using flow cytomytric analysis. The correlation between cell surface PDGFRA expression or EGFR expression and cell growth rate was analyzed using SPSS software.

### Bromodeoxyuridine (BrdU)-based Cell Proliferation Assay

Glioma cells were treated at the indicated conditions, and then incubated with or without 20 µM BrdU for 12 hrs at 37°C. The percentage of BrdU positive cell was assessed using BD Pharmingen™ BrdU Flow Kits in accordance with the manufacture’s instruction manual. Briefly, the cells were fixed/permeablized, treated with Dnase, labeled with anti-BrdU antibody and acquired on a flow cytometer.

### Immunocytochemistry Staining of Cultured Glioma Cells

Glioma cells were washed twice and fixed with 4% PFA for 30 min, followed by permeabilization with 0.1% Triton-X 100 for 30 min. The blocking solution containing 5% normal donkey serum in PBS was applied for 15 min. Primary antibodies against PDGFRA (clone αR1, BD), EEA-1 (polyclonal, Abcam), Rab11 (polyclonal, Abcam), Caveolin-1 (polyclonal, Abcam), Clathrin (polyclonal, Abcam) and Giantin (polyclonal, Abcam) were diluted in blocking solution and incubated overnight at 4°C. Immunoreactivity was done using fluorescently labeled secondary antibodies (1∶200) and visualized by confocal microscopy (Carl Zeiss, Germany). Co-localization analysis was performed using a ZEN2009 software based on Pearson’s correlation coefficient analysis which recognizes the colocalized pair by comparison pixel by pixel intensity.

### Immunohistochemistry Staining in Glioma Sections

Glioma tissues were surgically isolated and immediately fixed in 4% PFA, embedded in paraffin and cut into 5-µm sections. Staining was done was followed standard immunohistochemistry staining protocol using primary antibodies against PDGFRA (BD, USA), Cadherin (Abcam, UK), Tubulin (Sigma, USA), EEA-1 (clone 14/EEA1, BD), Rab11 (clone 47/Rab11, BD) Ki-67 (clone Ki-S5, Millipore) and fluorescently labeled secondary antibodies (1∶200). The images were acquired with Meta 510 confocal microscopy (Carl Zeiss, Germany) and fluorescent intensity was detected by ZEN2009 software. The extent of surface PDGFRA expression was calculated as the ratio between the mean PDGFRA intensity in plasma membrane and to mean PDGFRA intensity in cytosol, according to the formula: 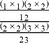. Where i_1_, i_2_ and i_3_ represent the intensities of whole cell, cytosol and nucleus, *a_1_*_,_ *a_2_* and *a_3_* represent the area of whole cell, cytosol and nucleus respectively.

### Total Internal Reflection Fluorescence Microscopy (TIRFM) Imaging

Glioma cells were seeded on MatTek dishes and stained for PDGFRA (clone αR1, BD) by the standard immunocytochemistry protocol. TIRFM images were acquired by a high-aperture 100× objective lens in an inverted epifluorescence microscope (Carl Zeiss). Before image acquisition, the penetration depth of the evanescent wave was determined by 3 µm fluorescent beads. To visualize the Cy2-labeled PDGFRA, the cells were excited using the 488-nm line of an argon laser and a 515 nm long pass emission filter. The images were collected by a CCD camera (COOLSNAP, Photometrics, UK), which was operated by the MetaMorph software (Molecular device, USA). Image analysis was done using ImageJ freeware (NIH, USA) and the numbers of PDGFRA clusters were calculated by using the cell counter plug-in.

### Statistical Analysis

The data are presented as averages ± SD. Evaluation of statistical significance was done by Student’s *t*-test for experiments allowing paired comparisons, or by one-way analysis of variance (ANOVA) with the Friedman test for multiple comparison.

## Supporting Information

Table S1 **Morphological diagnosis of glioma samples, age at diagnosis and survival period of the patients involved in this investigation.**
(PDF)
