# Supplementary material for: Glioma Cell Proliferation Controlled by ERK Activity-Dependent Surface Expression of PDGFRA
Source: PLoS One. 2014 Jan 29;9(1):e87281. doi: 10.1371/journal.pone.0087281 (PMC3906156; doi:10.1371/journal.pone.0087281)
Supplement: Table S1 — Morphological diagnosis of glioma samples, age at diagnosis and survival period of the patients involved in this investigation. (PDF) [file pone.0087281.s001.pdf]

Supplementary materials

Table S1. Morphological diagnosis of glioma samples, age at diagnosis and survival period of the patients involved in this investigation.

Sample No	Diagnosis	Age at diagnosis (Year)	Survival period (Year)
1	GBM	36	3.1
2	GBM	67	0.6
3	GBM	46	1.1
4	GBM	8	4.1
5	GBM	67	0.4
6	GBM	30	3.7
7	Astrocytoma II	29	6.2
8	Astrocytoma II	36	13
9	Astrocytoma II	60	1.8
10	Astrocytoma II	36	0.5
11	Astrocytoma II	22	3.5
12	GBM	47	0.3
13	Astrocytoma II	52	3.3
14	GBM	65	1.0

The survival period was calculated between the date of surgery and the date of death.
